# Enteroparasitism in Hard-to-Reach Community Dwellers: A Cross-Sectional Study in Ga West Municipality in Ghana

**DOI:** 10.1155/2020/8890998

**Published:** 2020-09-24

**Authors:** Enoch Aninagyei, Ruby Yirenkyi, Tanko Rufai, Margaretta Gloria Chandi

**Affiliations:** ^1^Department of Biomedical Sciences, School of Basic and Biomedical Sciences, University of Health and Allied Sciences, Ho, Volta Region, Ghana; ^2^Department of Molecular Biology and Biotechnology, University of Cape Coast, Cape Coast, Ghana; ^3^Ghana Health Service, Accra, Ghana; ^4^New Juabeng Municipal Health Directorate, Koforidua, Ghana; ^5^Ga North Municipal Health Directorate, Ofankor-Accra, Greater Accra Region, Ghana

## Abstract

Ga West Municipality in Ghana is predominantly rural with about forty-eight hard-to-reach communities. Several infectious diseases such as Buruli ulcer, tuberculosis, yaws, schistosomiasis, and malaria are prevalent in the municipality. However, the prevalence and characteristics of enteroparasites in the municipality are unknown. Therefore, this cross-sectional study determined the prevalence of enteroparasites in these hard-to-reach communities. Samples were collected from five communities, namely, Opah, Otuaplem, Dedeman, Onyansana, and Manchie. A total of 538 stool samples were collected from the community dwellers. Each sample was examined with eosin-saline wet preparation and formol-ether concentration technique. Body mass index, haemoglobin, and albumin concentrations were used to assess nutritional status. Seven different parasite species were identified in 178 community dwellers (33.1% prevalence (95% CI: 0.29–0.37)). The individual prevalence of the identified parasites was *Schistosoma mansoni* (13.4%), *Entamoeba histolytica* (7.2%), *Ascaris lumbricoides* (6.9%), *Giardia lamblia* (5.0%), hookworm (4.8%), *Strongyloides stercoralis* (4.8%), and *Balantidium coli* (1.6%). Among the 178 parasitized individuals, 68.0% were singly infected while 31.5% had dual parasitism. Significantly higher infections were associated with Onyansana dwellers (*p* = 0.019), participants aged 16–20 years (*p* = 0.006), unmarried participants (*p* < 0.001), those without formal education (*p* = 0.044), and crop farmers (*p* = 0.044). However, among the Akan tribe (*p* = 0.015), Christians (*p* = 0.03), and participants with higher incomes (*p* = 0.012), infections were found to be lower. Compared to monoparasitism, dual parasitism was significantly associated with underweight (17.8 vs. 20.3 kg/m^2^), anaemia (7.7 vs. 9.8 g/dL), and malnutrition (27.6 vs. 31.9 g/L of albumin concentration). These findings underscore the fact that the Ga West Municipality is heavily burdened with different species of enteroparasites. Therefore, education on personal hygiene to reduce parasitic infections must be intensified while implementing regular mass deworming exercise in the municipality.

## 1. Introduction

Ga West Municipality is one of the sixteen districts in the Greater Accra Region of Ghana. The municipality is 60% rural, 25% periurban, and 15% urban. It is made up of about 150 communities with Amasaman as its municipal capital [1]. It occupies a total land surface area of about 300 square kilometers with an estimated population of 262,742 as of 2015 [2]. Of the 150 communities in the municipality, about 48 communities (32%) are hard-to-reach. These hard-to-reach communities have been cut off from other communities by poor road infrastructure and defective bridges. In rainy seasons (mid-May to July), these roads are not motorable. These communities lack good health facilities with educational facilities only at the basic school level. Also, some basic social amenities are lacking. Potable water is lacking in almost all of these hard-to-reach communities except water from hand-operated wells and dug-outs in some “fortunate” communities. Residents rely mostly on rivers and streams for survival [3].

Some infectious diseases are very prevalent in the municipality, and residents in these hard-to-reach communities are mostly affected. Buruli ulcer, a disease caused by *Mycobacterium ulcerans*, is highly prevalent in these communities [1, 4] as well as yaws, a tropical disease caused by *Treponema pertenue* [5]. In these communities, tuberculosis, caused by *Mycobacterium tuberculosis* [6, 7], has also been found to be prevalent. Parasitic diseases such as malaria [8] and schistosomiasis [8, 9] are very common in the municipality, particularly in these poor communities.

Despite high prevalence of these infectious diseases in these communities, the burden of enteroparasites is unknown, hence scarcity of data in this regard. Therefore, this study was designed to determine the prevalence of enteroparasites in residents of these hard-to-reach communities in the Ga West Municipality of the Greater Accra Region of Ghana and also to determine some factors and clinical effects that are associated with enteroparasitism in parasitized individuals.

## 2. Methods

### 2.1. Study Design and Study Plan

This observational study, conducted between September 2019 and March 2020, was carried out to determine the prevalence of enteroparasites in selected communities in Ghana. Demographic indicators associated with parasitism were explored. Also, the effects of enteroparasitism on parasitized individuals were described. A list of hard-to-reach communities was obtained from the Ga West Municipal Health Administration.

### 2.2. Description of Study Areas

**Figure 1:**
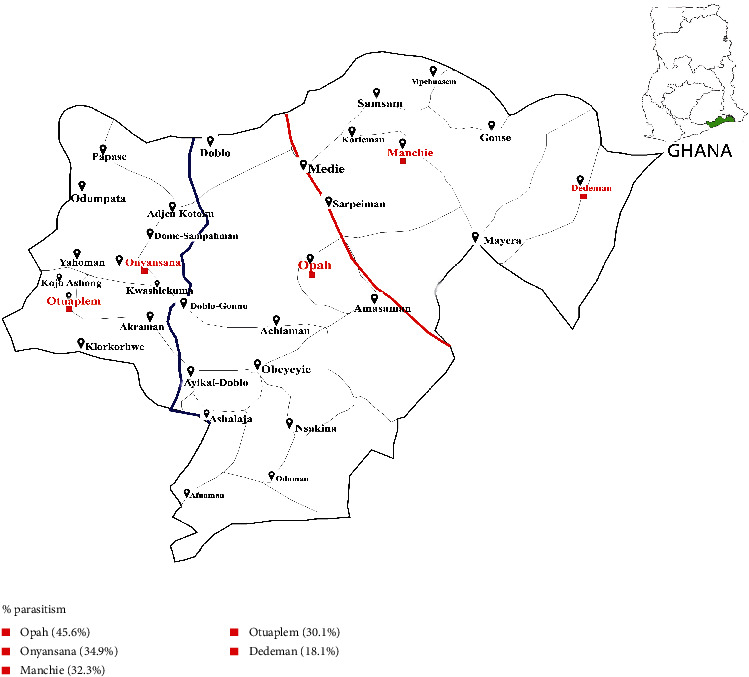
Map of Ga West Municipality, Ghana, showing study sites in red and prevalence of parasitism in study communities (the map is the authors' own production).

This study was done in hard-to-reach communities in the Ga West Municipality in the Greater Accra Region of Ghana. The communities were Opah, Otuaplem, Dedeman, Onyansana, and Manchie (Figure 1). These five communities were blindly selected from a list of about 48 communities in a simple random manner. From the municipal capital, Amasaman, where the municipal hospital was located, Opah, Manchie, Onyansana, Dedeman, and Otuaplem are about 16 km, 19 km, 20 km, 26 km, and 28 km away, respectively. These communities are connected to the municipal capital by poor road networks. By road, it takes approximately 45 minutes to travel from Opah to Amasaman and about 2 hours to travel from the other four communities to Amasaman. These are predominantly farming communities with uncoordinated sand winning activities. None of these communities has either a hospital or a clinic. In Ghana, the basic health care facility is community-based health services (CHPS). These hard-to-reach communities do not have a CHPS compound. Residents travel to nearby communities to assess community health services.

### 2.3. Selection of Households, Study Participants, and Stool Sample Collection

With the help of the community leaders, each household was numbered after which 10% of households were randomly selected to select participants from. Consent to include the household in the study was sought from the heads of the household. Consent to participate was also obtained from all participants and assent for participants under 18 years obtained from their guardians. After obtaining consent, the number of household occupants was noted and half of that number, to the nearest whole number, was sampled. A number of stool containers corresponding to half the number of household occupants were given to the heads of the household to be given, randomly, to the occupants for provision of about 4 g stool samples. The heads of the household were taken through proper stool sample collection protocols. Briefly, during defecation, the prelabelled stool container was opened, with the aid of the accompanying plastic disposable spoon; at least five spoonfuls were collected into the container. In the case of watery stool, containers were filled, using the disposable plastic funnel provided, to about one-third, without soiling the container. After sample collection, household heads ensured that the hands were properly washed and disinfected with a hand sanitizer containing 70% alcohol. Stool samples were provided by participants early in the morning, placed in a prelabelled biohazard bag, and kept in a biohazard container. Samples were collected by 10 am and sent to the laboratory.

#### 2.3.1. Sample Size Determination

The minimum number of samples collected from the study sites was determined using the formula *n* = *z*^2^*p*(1 − *p*)/*d*^2^, where *n* is the sample size, *p* = prevalence of enteroparasitism in Ga West Municipality, *z* = confidence level at 95% (standard value of 1.96), and *d* = margin of error at 5% (standard value of 0.05) [10]. Prevalence of enteroparasitism in Ga West is unknown, so prevalence was estimated at 50%. The sample size was calculated to be 384. To cater for missing and incomplete data, the sample size was increased by 10%. Therefore, the minimum sample size was 423.

#### 2.3.2. Inclusion and Exclusion Criteria

Households included in this study were household with available heads to consent to the study. Also, participants included in the study were household occupants over five years old and those that have stayed in the community for at least a year. On the other hand, residents that have dewormed, using a Ministry of Health-approved dewormer, were excluded. Finally, household occupants that dissented participation and individuals that declined blood sample collection were also excluded.

### 2.4. Stool and Blood Sample Collection and Collection of Relevant Study Information

Early in the morning of sample provision, the research team was in the household to collect stool and blood samples and then administer a questionnaire to obtain some information from the study participant. Information obtained from the participants was age, gender, highest education, and occupation. Stool samples were divided into two equal parts; one part was fixed in 10% formol-saline solution and the other left unfixed. A sample pair was kept at ambient temperature prior to arrival in the Ga North Municipal laboratory. Whole blood was collected from a prominent vein at the antecubital fossa region of the forearm. Prior to blood collection, the selected area to perform venepuncture was disinfected with 70% alcohol and allowed to air dry before performing venepuncture. Five mL of whole blood was collected into the EDTA tube, mixed gently, and kept on ice till plasma was separated into another tube.

### 2.5. Nutritional Assessment

Body mass index (BMI), haemoglobin, and albumin concentrations were used to assess the nutritional status of the participants as previously used [11, 12].

#### 2.5.1. Determination of Body Mass Indices

BMI was calculated by dividing the weight in kilograms (kg) by the square of height in meters. Height was taken by a Seca 213 portable stadiometer (New Zealand), and body weight was taken by an Omron digital weighing scale (Omron, Kyoto, Japan). Height was taken barefooted, and weight was also taken in light cloths. Participants with BMI in kg/m^2^ < 18.5, 18.5-24.9, 25.0-29.9, and >30.0 were classified as underweight, normal, overweight, and obese, respectively, according to [13].

#### 2.5.2. Determination of Haemoglobin Concentration

Haemoglobin concentration was determined by using a hand-held haemoglobinometer (URIT-12, Guangzhou, China). Prior to first use, the meter was calibrated using the accompanying calibration chip. The haemoglobin concentration was determined following the manufacturer's instruction. Briefly, a strip was inserted into the meter till the drop of blood sign appears on the screen; a drop of well-mixed anticoagulated blood was dropped on the sample receptacle portion of the strip. A result was obtained in about 5 seconds. Haemoglobin levels < 8.0 g/dL, 8.0-10.9 g/dL, 11.0–11.9 g/dL, and >12.0 g/dL were classified as severe anaemia, moderate anaemia, mild anaemia, and nonanaemic, respectively, according to a World Health Organization publication on haemoglobin concentrations for the diagnosis of anaemia and assessment of severity [14].

#### 2.5.3. Determination of Albumin Concentration

The albumin concentration was measured by a PKL-125 Italia fully automated chemistry analyser using an ELItech albumin endpoint reagent (France) based on the reaction: albumin + bromocresol green (BCG) → albumin‐BCG complex.

The albumin-BCG complex absorbs maximally at 630 nm. Albumin values below mean minus 1SD and above mean minus 2SD indicated malnourishment while values below mean minus 2SD indicated severe malnourishment. Classification was adopted from Omitola et al. [15].

### 2.6. Microscopic Detection of Enteroparasites

#### 2.6.1. Detection of Enteroparasites Using Eosin-Saline Wet Preparation

On the same day of sample arrival in the laboratory, wet preparation was made to determine viable parasites. The stool sample was well mixed in its container and a drop transferred onto a microscope slide. The sample was emulsified on the slide using equal volume of normal saline and eosin mixture. The emulsified stool sample was examined using ×10 and ×40 objective of the microscope. Parasites were identified using their characteristic shape and motility [16].

#### 2.6.2. Detection of Enteroparasites Using Formol Ether Concentration (FEC) Method

Stool samples were emulsified with saline (0.9% NaCl) into a homogenous mixture. One mL of the homogenate was poured into a centrifuge tube after which 4.0 mL of formol-saline reagent was added. The mixture was vortexed vigorously before another 3.0 mL of 10% formol-saline was added. The content was mixed and sieved through a nonabsorbent sieve (3- or 4-folds). Subsequently, 3 mL of diethyl ether (Honeywell, USA: bp: 34.6°C, mp: -116.3°C, mm: 74.12 g/mol) was added and vortexed vigorously. The content was centrifuged at 3,000 rpm for 2 minutes. Supernatant was discarded, and iodine-mixed deposits were examined at ×10 and ×40. Parasite ova were identified using characteristic shapes [16].

### 2.7. Deworming of the Infected Individuals

Infected individuals were referred to nearby hospitals for treatment according to World Health Organization guidelines [17, 18]. Whereas praziquantel (20 mg/kg) was used to treat for *S. mansoni*, the other parasites were treated with albendazole (400 mg). This treatment protocol was reviewed and approved by the Ghana Health Service Ethical Review Committee (Reference: GHS-REC002/03/18).

### 2.8. Outcome of the Study

The study reported overall and individual prevalence of enteroparasites identified in the study sites. The morphological characterization of the parasites was reported. Also, the frequencies of parasitism in the various age ranges, gender category, educational status, and occupation were reported. Finally, the effects of the parasites on nutritional status determined by body mass index, haemoglobin, and albumin concentrations were also reported.

### 2.9. Data Analysis

Frequencies were presented using percentages while prevalence was calculated based on the number of individuals parasitized divided by the total number of individuals tested in each category. Parasitism was classified as single, dual, and triple parasitism when one, two, or three different parasites were found in an individual. A chi-squared test was used to examine the association between infectious status and the independent variables while the logistic regression model was used as a post hoc analysis tool. Statistical analysis was done by SPSS Version 24 (Chicago, IL, USA). *p* value of less than 0.05 was considered statistically significant.

## 3. Results

### 3.1. Demographic Characteristics of the Study Participants

Study participants (*n* = 538) were selected from five hard-to-reach communities in Ga West Municipality, namely, Opah (*n* = 149), Otuaplem (*n* = 136), Dedeman (*n* = 105), Onyansana (83), and Manchie (*n* = 65). Majority of the participants (61.5%) were males while the modal age range was 16–20 years. A little over 50% of the participants has never had formal education or has had only primary education. It was also found that 62.8% of the participants were farmers while the rest were either unemployed, pupils in primary school, petty traders, or sand winners. Of the 338 (62.8%) farmers, 61.8% were crop farmers and 13.9% were poultry farmers while 24.3% were livestock farmers (Table 1).

### 3.2. Prevalence of Enteroparasitism

The overall prevalence of enteroparasitism in the hard-to-reach communities in the municipality was 33.1% (178/538) (95% CI: 0.29–0.37). The most prevalent community was Opah (45.6%) while Dedeman was the least prevalent (18.1%). The other three communities were almost equiprevalent (30.1–34.9%) (Figure 1). Prevalence of parasitism among males and females was 55.5% and 19.0%, respectively. Community dwellers up to 20 years were disproportionately parasitized (76.4%) with individuals aged 16–20 years heavily parasitized (62.7%). Again, community dwellers with no or only primary education were most parasitized (64.0%) while those educated up to the postsecondary level were least parasitized (13.6%). The prevalence of enteroparasitism was higher among sand winners, farmers, and primary school pupils (36.1%, 34.9%, and 32.9%, respectively). Unemployed and petty traders were least parasitized even though the prevalence was higher in the unemployed (26.8%) than petty traders (16.7%). Among the farmers, the prevalence of parasitism was higher in crop farmers (46.9%) than poultry (14.6%) and livestock (15.8%) farmers (Table 1).

### 3.3. Association of Infection Status with Study Variables

A chi-squared tool was used to test the association of infection status with independent variables. Whereas residential community (*χ*^2^ = 21.9, *p* < 0.0001), age of participants (*χ*^2^ = 89.9, *p* < 0.0001), marital status (*χ*^2^ = 67.9, *p* < 0.0001), tribe (*χ*^2^ = 61.8, *p* < 0.0001), religious affiliation (*χ*^2^ = 91.1, *p* < 0.0001), average monthly income (*χ*^2^ = 38.4, *p* < 0.0001), educational level of participants (*χ*^2^ = 37.1, *p* < 0.0001), and farming type (*χ*^2^ = 36.9, *p* < 0.0001) were associated with infection status, gender (*χ*^2^ = 1.1, *p* = 0.301) and occupation of the participants (*χ*^2^ = 5.1, *p* = 0.278) were not associated (Table 1). Subsequently, gender and participants' occupation types were excluded from logistic regression analysis. Within the set of independent variables, logistic regression analysis indicated significantly higher infections in Onyansana (*p* = 0.019), 16–20 years of age range (*p* = 0.006), unmarried participants (*p* < 0.001), those without formal education (*p* = 0.044), and crop farmers (*p* = 0.044). However, among the Akan tribe (*p* = 0.015), Christians (*p* = 0.03), and participants with higher incomes (*p* = 0.012), infections were found to be lower (Table 2).

### 3.4. Characterization and Prevalence of Enteroparasites

A cumulative total of 236 infections comprising seven parasite species were identified in 178 community dwellers. Both protozoa and helminths were identified. Three different protozoa were identified, namely, *Giardia lamblia* (*n* = 27, 5.0%), *Entamoeba histolytica* (*n* = 39, 7.2%), and *Balantidium coli* (*n* = 9, 1.6%). Also, four different helminths were identified. They were *Ascaris lumbricoides* (*n* = 37, 6.9%), *Schistosoma mansoni* (*n* = 72, 13.4%), hookworm (*n* = 26, 4.8%), and *Strongyloides stercoralis* (*n* = 26, 4.8%). Among the 178 parasitized individuals, 121 (68.0%) were singly infected with either *G. lamblia*, *E. histolytica*, *B. coli*, *A. lumbricoides*, *S. mansoni*, hookworm, or *S. stercoralis*. While 56 (31.5%) of the community dwellers had dual parasitism, the common double parasitism was *A. lumbricoides* and *S. mansoni* (3.3%) followed by *A. lumbricoides* and hookworm (2.4%). However, *B. coli* and *E. histolytica* dual parasitism was rare (0.7%). Only one (0.18%) triple parasitism comprising *G. lamblia*, *E. histolytica*, and *S. stercoralis* was identified (Table 3).

### 3.5. Comparing Nutritional Status of Parasitized and Nonparasitized Community Dwellers

Over 60% of parasitized participants were underweight while a little over 10% of the nonparasitized participants were underweight. Again, 68.0% of the parasitized participants had various forms of anaemia while 33.8% of the nonparasitized participants had various forms of anaemia. Finally, 64.5% of the parasitized participants were either malnourished or severely malnourished while 23.6% of the nonparasitized were malnourished, but none was severely malnourished (Table 4).

### 3.6. Frequency of Association of Enteroparasites with Nutritional Statuses

Three parasites (*S. mansoni* (*n* = 69; 63.3%), hookworm (*n* = 27; 24.8%), and *E. histolytica* (*n* = 13; 11.9%)) were identified in 109 participants that were underweight. With the exception of *B. coli*, the other parasites (*A. lumbricoides*, *E. histolytica*, *G. lamblia*, hookworm, *S. mansoni*, and *S. stercoralis*) were identified in participants with various forms of anaemia. Hookworm (*n* = 19; 61.3%), *G. lamblia* (*n* = 7; 22.6%), and *S. mansoni* (*n* = 5; 16.1%) were identified in severely anaemic study participants. Together with hookworm and *S. mansoni*, *E. histolytica* and *A. lumbricoides* were identified in moderately anaemic participants. Again, in mildly anaemic participants, hookworm (*n* = 11, 35.5%), *G. lamblia* (*n* = 9, 29.0%), *S. stercoralis* (*n* = 7, 22.6%), and *E. histolytica* (*n* = 4, 12.9%) were identified. Finally, while *G. lamblia* (30.6%), *A. lumbricoides* (29.2%), hookworm (26.4%), and *E. histolytica* (13.9%) were identified in malnourished participants, *S. stercoralis* (39.5%), hookworm (25.6%), *G. lamblia* (23.3%), and *B. coli* (11.6%) were seen in severely malnourished participants (Table 5).

### 3.7. Impact of Parasitism on Nutritional Statuses

The mean BMI of monoparasitized individuals was significantly higher than individuals with dual parasitism (20.3 vs. 17.8; *p* = 0.035). Similarly, the mean haemoglobin concentration of monoparasitized individuals was significantly higher than dual parasitized individuals (9.8 vs. 7.7; *p* = 0.018). Also, mean albumin concentration in monoparasitized individuals was higher than the mean concentration obtained for dual parasitism (31.9 vs. 27.6; *p* = 0.042). Surprisingly, the BMI of the only triple parasitized individual was higher than the mean values found in mono- and dual parasitism. Again, the haemoglobin concentration in triple parasitism was higher than the mean haemoglobin concentration in mono- and dual parasitism, but the albumin concentration for triple parasitism was higher than dual parasitism but lower than monoparasitism (Table 6).

## 4. Discussion

This study reports, for the first time, the prevalence of parasitic infections in the Ga West Municipality in the Greater Accra Region of Ghana. In this study, the overall parasitism in hard-to-reach communities in the municipality was 33.1%. In all, three protozoa, *Giardia lamblia*, *Entamoeba histolytica*, and *Balantidium coli*, and four helminths, three geohelminths (*Ascaris lumbricoides*, *Strongyloides stercoralis*, and hookworm), and one water-transmitted parasite, *Schistosoma mansoni*, were identified. Identification of these enteroparasites in hard-to-reach residents was consistent with previous studies that also identified protozoa and helminths in rural settings [19–24].

It is noteworthy that this study is the first to identify *B. coli* infections in humans in Ghana. However, a previous study in 2018 detected *B. coli* in commonly consumed vegetables collected from the Eastern Region of Ghana [25], a region that shares direct boundary with the Ga West Municipality. The prevalence of *B. coli* was 1.7% (9/538); five infections were single parasitism while four were seen together with *E. histolytica.* These parasites were identified in two livestock-rearing communities, namely, Opah and Onyansana. Pigs are known to be a reservoir of *B. coli*, and humans become infected through direct or indirect contact with pig droppings [26]. As observed in this study, *B. coli* is largely prevalent in rural areas in developing countries. Due to the possibility of pig and human fecal matter-containing rivers and streams [26], people who do not reside in pig-rearing communities may also be at risk of balantidiosis. *B. coli* is highly prevalent in Latin America, the Philippines, Papua New Guinea, and areas of the Middle East [27, 28]. The prevalence of *B. coli* in these regions is close to 29% in pig and other farmers [29, 30]. However, *B. coli* infection has the potential for worldwide distribution [30]. It is not too clear how *B. coli* was transported into Ghana, but it is possible that the parasite is prevalent in pig farmers in Ghana, as published previously [25], but lack of surveillance activities for the parasites makes it appear that it does not exist in Ghana. *B. coli* could be an emerging parasitic infection in rural Ghana where pigs are being reared.

This study found that males, residents less than 20 years, individuals with no or only primary education as well as pupils, sand winners, and crop farmers were disproportionally infected. Young adult males have been found to be at higher risk of parasitic infections due to their frequent outdoor activities that keep them in constant contact with infected soil [31–33]. Again, in rural Ghana, proper toilet facilities have been found to be lacking with common open defecation [34]. This unhygienic practice contaminates the soil with faeces containing parasite ova and cyst. This also makes land tillers such as crop farmers and sand winners highly vulnerable to parasitic infections. Again, this study found half (50%) of the schoolchildren to be infected with various parasites. This finding is consistent with previous studies that found schoolchildren to be at higher risk of parasitism due to their constant contact with the soils [35–39] coupled with poor hygienic environment in rural areas as well as unhygienic practices such as poor hand-washing practices, walking barefooted, and touching their mouths with unclean hands. Education has also been found to be associated with parasitic infections with low education associated with high rates of infections. In this study, 64.0% of the study participants with no or only primary education were most parasitized. This is because well-educated people are able to understand and adhere to parasite control practices [40] that have the ultimate outcome of reducing parasitic infections. Also, lack of potable water in the study communities made the community members depend on streams and rivers. This led to high prevalence of *S. mansoni* (13.4%) with males between 8 and 20 years predominantly infected.

This study found 31.5% of coparasitism in the community dwellers. The most common dual parasitism was *A. lumbricoides/S. mansoni* (10.0%), *A. lumbricoides/*hookworm (7.3%), and *G. lamblia/E. histolytica* (5.0%). Comparing to monoparasitism, dual parasitism was significantly associated with various degrees of anaemia, underweight, and moderate to severe malnourishment. This study, though confirming separate earlier reports [36, 37, 41], associated dual parasitism to underweight, anaemia, and malnourishment in the same parasitized individuals. Persistent parasitism is suggested to cause stunted growth due to the competition of the parasites with the host for essential nutrients [38, 39, 42]. Again, parasites such as *S. mansoni*, hookworm, and *E. histolytica* are known to impact anaemia to their host. *E. histolytica* trophozoites have been shown to depend on iron for their metabolism, reproduction, and survival inside the host [43] while *S. mansoni* has been found to cause anaemia through inflammatory response [35]. Hookworm is also known to cause iron deficiency anaemia by competing with the host for iron [44]. The clinical effects of these parasites on their hosts call for intense screening of residents of hard-to-reach communities for enteric parasites and also implementation of regular mass drug administration exercises to reduce enteroparasitism in these communities.

## 5. Conclusion

This study reports, for the first time, the prevalence and microscopic characterization of enteroparasites in hard-to-reach communities in the Ga West Municipality, Ghana. *G. lamblia*, *E. histolytica*, *B. coli*, *A. lumbricoides*, *S. stercoralis*, hookworm, and *S. mansoni* were identified at different frequencies. In this study, community dwellers above 5 years were examined due to difficulty in getting stool samples from children under 5 years. Assessing the prevalence of enteroparasites in children under 5 will complement this study. Again, this study identified *B. coli* for the first time in humans in Ghana. The number of *B. coli* infections identified was not enough to describe their clinical significance on their host. But it was found that the stool samples that contained them were loose to watery. Dual parasitism was found to be associated with underweight, anaemia, and malnutrition. Education on personal hygiene with the aim of prevention of parasitic infections must be intensified in the municipality. Mass deworming exercise in the entire municipality is also recommended. After implementation of these control measures, future studies to reassess the persistence of enteroparasites in these communities are recommended.

## Figures and Tables

**Table 1:** Association of prevalence of enteroparasitism with sociodemographic variables.

Demographic characteristics	Number of samples	Prevalence of parasitism		Chi-squared test	
		Number of infected individuals	%	Chi-squared value (*χ*^2^)	*p* value
Resident community				21.9	<0.0001
Opah	149 (27.7%)	68	45.6		
Otuaplem	136 (25.3%)	41	30.1		
Dedeman	105 (19.5%)	19	18.1		
Onyansana	83 (15.4%)	29	34.9		
Manchie	65 (12.1%)	21	32.3		
Gender				1.1	0.301
Males	331 (61.5%)	115	55.5		
Females	207 (38.5%)	63	19.0		
Age range (years)				89.9	<0.0001
6–10	57 (10.6%)	30	52.6		
11–15	118 (21.9%)	37	31.5		
16–20	110 (20.4%)	69	62.7		
21–25	79 (14.7%)	21	26.5		
26–30	89 (16.5%)	9	10.1		
31–35	41 (7.6%)	7	17.1		
36–40	25 (4.6%)	2	8.0		
>40	19 (3.5%)	3	15.8		
Marital status				67.9	<0.0001
Single	188 (34.9)	105	55.9		
Married	294 (54.6)	63	21.4		
Widowed	18 (3.3)	3	16.7		
Divorced	38 (7.1)	7	18.4		
Tribe				61.8	<0.0001
Akan	59 (11.0)	8	13.6		
Ga	87 (16.2)	32	36.8		
Ewe	279 (51.9)	71	25.4		
Dagomba	33 (6.1)	17	51.5		
Fulani	71 (13.2)	48	67.6		
Others^a^	9 (1.7)	2	22.2		
Religion				91.1	<0.0001
Christianity	282 (52.4)	66	23.4		
Islamic	192 (35.7)	91	47.4		
Traditional	49 (9.1)	19	38.8		
Others^b^	15 (2.8)	2	13.3		
Average monthly income^c^				38.4	<0.0001
0–500 (0–90)	120 (28.9)	56	46.7		
501–1000 (91–181)	82 (19.8)	33	40.2		
1001–1500 (182–272)	78 (18.8)	29	37.2		
1501–2000 (273–363)	59 (14.2)	13	22.0		
>2000 (>364)	76 (18.3)	9	11.8		
Highest education				37.1	<0.0001
None	93 (17.3%)	53	56.9		
Primary	181 (33.6%)	61	33.7		
Junior high	153 (28.4)	33	21.6		
Senior high	89 (16.5%)	28	31.5		
Postsenior high	22 (4.1%)	3	13.6		
Occupation				5.1	0.278
Unemployed	41 (7.6%)	11	26.8		
Pupil	82 (15.2%)	27	32.9		
Petty trading	30 (5.6%)	5	16.7		
Sand winning	47 (8.7%)	17	36.1		
Farming	338 (62.8%)	118	34.9		
Type of farming				36.9	<0.0001
Crop	209 (61.8%)	98	46.9		
Poultry	47 (13.9%)	7	14.6		
Livestock	82 (24.3%)	13	15.8		

^a^Other tribes were Frafra (*n* = 1), Guan (*n* = 2), Hausa (*n* = 3), and Krobo (*n* = 3); ^b^Buddhist (*n* = 2), Afrikanian mission (*n* = 4), unification movement (*n* = 4), and atheist (*n* = 5); ^c^income quoted in Ghana cedi ($), exchange rate of 1 USD to 5.5 Ghana cedis.

**Table 2:** Logistic regression analysis of significant associations of enteroparasitism with study variables.

Study variables	*β*	S.E.	Wald	df	Sig.	Exp (*β*)
Resident community						
Opah	0.124	0.463	0.071	1	0.789	1.132
Otuaplem	-0.025	0.515	0.002	1	0.961	0.975
Dedeman	0.810	0.484	2.798	1	0.094	2.247
Onyansana	1.001	0.427	5.506	1	0.019^∗^	2.722
Manchie (reference)						
Age range (years)						
6–10	-2.067	4.537	0.208	1	0.649	0.127
11–15	-2.973	1.654	3.228	1	0.072	0.051
16–20	3.375	1.223	7.615	1	0.006^∗^	29.232
21–25	0.972	1.235	0.619	1	0.431	2.643
26–30	-0.690	1.262	0.299	1	0.584	0.501
31–35	-1.390	1.718	0.655	1	0.418	0.249
36–40	-0.920	1.468	0.393	1	0.531	0.398
>40 (reference)						
Marital status						
Single	5.933	1.606	13.644	1	<0.001^∗^	377.362
Married	0.380	1.061	0.128	1	0.721	1.462
Widowed	0.980	1.227	0.638	1	0.424	2.665
Divorced (reference)						
Tribe						
Akan	-2.964	1.213	5.969	1	0.015^∗^	0.052
Ga	-2.169	1.281	2.865	1	0.090	0.114
Ewe	-2.175	1.113	3.820	1	0.051	0.114
Dagomba	-1.306	1.256	1.081	1	0.298	0.271
Fulani	-1.914	1.139	2.822	1	0.093	0.147
Others (reference)						
Religion						
Christianity	-1.006	0.464	4.709	1	0.030^∗^	0.366
Islamic	1.258	0.694	3.285	1	0.070	3.519
Traditional	-1.680	0.985	2.911	1	0.088	0.186
Others (reference)						
Average monthly income						
0–500 (0–90)	-1.715	1.004	2.915	1	0.088	0.180
501–1000 (91–181)	0.118	0.428	0.075	1	0.784	1.125
1001–1500 (182–272)	-1.719	0.681	6.363	1	0.012^∗^	0.179
1501–2000 (273–363) (reference)						
Highest education						
None	-1.136	0.564	4.056	1	0.044^∗^	0.321
Primary	-0.311	0.692	0.202	1	0.653	0.733
Junior high	-0.459	0.809	0.321	1	0.571	0.632
Senior high	-0.732	0.534	1.878	1	0.171	0.481
Postsenior high (reference)						
Type of farming						
Crop	-2.474	1.226	4.069	1	0.044^∗^	0.084
Poultry	-1.611	0.910	3.138	1	0.076	0.200
Livestock	0.443	1.205	0.135	1	0.713	1.558
Not applicable (reference)						

^∗^Significantly different variable.

**Table 3:** Prevalence of single and coparasitism in study participants.

Parasite	Number of participants	%
Monoparasitism	121	22.5
*Giardia lamblia*	12	2.2
*Entamoeba histolytica*	25	4.6
*Balantidium coli*	5	0.9
*Ascaris lumbricoides*	6	1.1
*Schistosoma mansoni*	47	8.7
Hookworm	6	1.1
*Strongyloides stercoralis*	20	3.7
Dual parasitism	56	10.4
*G. lamblia*+*E. histolytica*	9	1.7
*B. coli*+*E. histolytica*	4	0.7
*A. lumbricoides*+hookworm	13	2.4
*S. stercoralis*+*G. lamblia*	5	0.9
*A. lumbricoides*+*S. mansoni*	18	3.3
*S. mansoni*+hookworm	7	1.3
Triple parasitism	1	0.18
*G. lamblia*+*E. histolytica*+*S. stercoralis*		

**Table 4:** Anthropometric parameters and nutritional statuses of infected and noninfected study participants.

Anthropometric features	Number of infected individuals	Number of noninfected individuals
Body mass index (kg/m^2^)		
Underweight (<18.5)	109 (61.2%)	37 (10.3%)
Normal (18.5–24.9)	55 (30.9%)	193 (53.6%)
Overweight (25.0–29.9)	11 (6.2%)	66 (18.3%)
Obese (>30.0)	3 (1.7%)	64 (17.8%)
Anaemia assessment		
Severe anaemia (Hb < 8.0 g/dL)	31 (17.4%)	21 (5.8%)
Moderate anaemia (Hb 8.0-10.9 g/dL)	59 (33.1%)	80 (22.2%)
Mild anaemia (11.0–11.9 g/dL)	31 (17.4%)	95 (26.4%)
Nonanaemic (Hb > 12.0 g/dL)	57 (32.0%)	164 (45.6%)
Serum albumin levels		
Values above mean minus 1SD (normal)	63 (35.4%)	275 (76.4%)
Values below mean minus 1SD and above mean 2SD (malnourished)	72 (40.4%)	85 (23.6%)
Values below mean minus 2SD (severely malnourished)	43 (24.1%)	0 (0.0%)

In the infected group, mean albumin concentration was 41.4 g/L while the standard deviation (SD) was 6.7 g/L. Mean minus 1SD was 34.7 g/L while mean minus 2SD was 28.0 g/L. In the noninfected group, mean albumin was 44.3 g/L and SD was 5.3 g/L. Mean minus 1SD was 39.0 g/L while mean minus 2SD was 33.7 g/L.

**Table 5:** Distribution of enteroparasites in underweight, anaemic, and malnourished participants.

Nutritional status	Associated parasites	Frequency of association with nutritional status
Underweight (*n* = 109)	*S. mansoni*	69 (63.3%)
	Hookworm	27 (24.8%)
	*E. histolytica*	13 (11.9%)
Severe anaemia (*n* = 31)	Hookworm	19 (61.3%)
	*G. lamblia*	7 (22.6%)
	*S. mansoni*	5 (16.1%)
Moderate anaemia (*n* = 59)	Hookworm	22 (37.3%)
	*E. histolytica*	19 (32.2%)
	*S. mansoni*	9 (15.3%)
	*A. lumbricoides*	9 (15.3%)
Mild anaemia (*n* = 31)	Hookworm	11 (35.5%)
	*G. lamblia*	9 (29.0%)
	*S. stercoralis*	7 (22.6%)
	*E. histolytica*	4 (12.9%)
Malnourished (*n* = 72)	*G. lamblia*	22 (30.6%)
	*A. lumbricoides*	21 (29.2%)
	Hookworm	19 (26.4%)
	*E. histolytica*	10 (13.9%)
Severely malnourished (*n* = 43)	*S. stercoralis*	17 (39.5%)
	Hookworm	11 (25.6%)
	*G. lamblia*	10 (23.3%)
	*B. coli*	5 (11.6%)

**Table 6:** Differences in haemoglobin, albumin levels, and BMI in categories of parasitism.

Nutritional indicator	Monoparasitism (*n* = 121)	Dual parasitism (*n* = 56)	Triple parasitism (*n* = 1)	*p* value^∗^
Body mass index	20.3 kg/m^2^^∗^	17.8 kg/m^2^^∗^	24.5 kg/m^2^	0.035
Haemoglobin concentration	9.8 g/dL^∗^	7.7 g/dL^∗^	10.7 g/dL	0.018
Albumin concentration	31.9 g/L^∗^	27.6 g/L^∗^	28.5 g/L	0.042

^∗^ *p* values were determined by a *t*-test. Analysis determined differences in nutritional indicators in mono- and dual parasitism only excluding triple parasitism due to its single occurrence.

## Data Availability

All data obtained in this study have been presented in the manuscript.
